# Pancreatic adenocarcinoma-associated polymyositis treated with corticosteroids along with cancer specific treatment: case report

**DOI:** 10.1186/1471-230X-11-33

**Published:** 2011-04-07

**Authors:** John Syrios, Georgios Kechagias, Ioannis D Xynos, Maria N Gamaletsou, Aristea Papageorgiou, George Agrogiannis, Nicolas Tsavaris

**Affiliations:** 1 Department of Pathophysiology, Laiko General Hospital, Medical School, National and Kapodistrian University of Athens, Mikras Asias 75, 11527 Athens, Greece; 2 1st Department of Pathology, Medical School, National and Kapodistrian University of Athens, Athens, Mikras Asias 75, 11527 Athens, Greece

## Abstract

**Background:**

Adenocarcinoma of the pancreas only rarely is associated with inflammatory myopathy. In this setting, polymyositis may be treated with glucocorticoids in combination with cancer specific treatment.

**Case presentation:**

We present the case of a 52-year-old man with stage IIA pancreatic tail adenocarcinoma who underwent surgical treatment and six months into therapy with gemcitabine he developed symmetrical, painful, proximal muscle weakness with peripheral oedema. Re-evaluation with imaging modalities, muscle histology and biochemistry conferred the diagnosis of polymyositis associated with pancreatic cancer progression. The patient was treated with glucocorticoids along with gemcitabine and erlotinib which resulted in complete remission within six months. He remained in good health for a further six months on erlotinib maintenance therapy when a new computer tomography scan showed pancreatic cancer relapse and hence prompted 2^nd ^line chemotherapy with gemcitabine.

**Conclusions:**

Polymyositis associated with pancreatic cancer may respond to glucocorticoids along with cancer specific treatment.

## Background

In developed countries, pancreatic adenocarcinoma is the fourth leading cause of cancer death, with an overall 5-year survival rate of less than 10% [1] and the incidence appears to be increasing. Despite the advances in chemotherapy, particularly gemcitabine, and the development of new tyrosine kinase inhibitors, such as erlotinib (Tarceva) an epidermal growth factor receptor (EGFR) inhibitor, the prognosis for patients with pancreatic cancer is dismal[1].

An association between malignancy and inflammatory myopathy was suspected as early as 1916, with adenocarcinomas of the cervix, lung, ovaries, pancreas, bladder, and stomach accounting for approximately 70 percent of the cancers associated with inflammatory myopathies[2]. On the other hand, patients with inflammatory myopathies, which commonly include dermatomyositis and polymyositis, have a clearly higher risk of cancer than the general population. Moreover, when inflammatory myopathies present with a significant weakness at diagnosis, they carry an unfavorable impact on prognosis[2-4].

Herewith we present a case of polymyositis complicating the physical history of a patient with pancreatic adenocarcinoma on treatment with gemcitabine who responded well to glucocorticoids along with cancer specific treatment.

## Case presentation

In March 2009, a 52-year-old Caucasian man, smoker 30 pack/y, with type II diabetes presented with a recent history of recurrent acute pancreatitis and significant weight loss (15 kg over 3 mo). Computer Tomography (CT) examination revealed a solitary mass lesion in the pancreatic tail and the patient subsequently underwent distal pancreatectomy coupled with splenectomy. Pathologic examination of the resected specimens conferred the diagnosis of a poorly differentiated adenocarcinoma which was locally invasive to the peripancreatic adipose tissue. Lymph nodes were negative and surgical margins were clear (T.N.M. stage IIA). The patient was treated with sequential adjuvant chemotherapy, six cycles of gemcitabine (1000 mg/m2) on days 1, 8, 15 and every 28 d with a steady decline of CA 19.9 levels. Six months into treatment with gemcitabine he developed symmetrical, painful, proximal muscle weakness in the upper and lower limbs with peripheral oedema and significant pain. The symptoms were severe enough to have confined him to a wheelchair.

**Figure 1:**
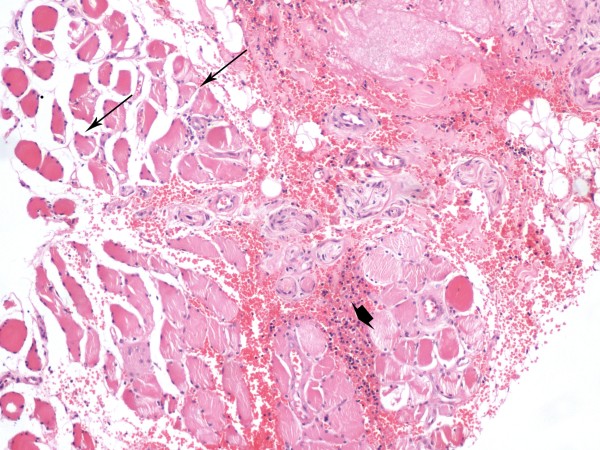
**Histological findings of muscle biopsy**. Muscle biopsy showing diffuse degenerative changes with angular fibers (arrows) and variation in muscle fiber size. Mononuclear inflammatory cells consisting of lymphocytes are present, surrounding individual non necrotic fibers (arrowhead). Some foci of fat infiltration and connective tissue septa thickening can also be seen. H&E stain, 100 × original magnification.

**Figure 2:**
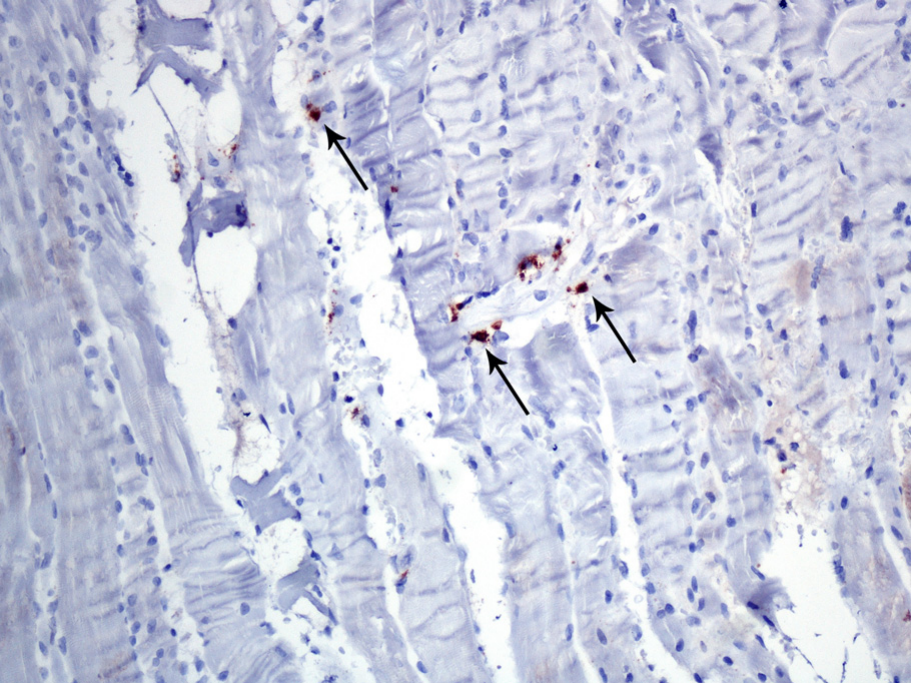
**CD8 immunohistochemical staining**. Immunohistochemical stainining for CD8 expressing lymphocytes. Arrows are indicating sparse infiltrates of intramycial CD8-positive cells. Brown diaminobenzidine staining, 200 × original magnification).

On readmission to hospital physical signs and history suggested the diagnosis of polymyositis. Aspartate aminotrasferase (AST) was 103 U/L (normal 5-40), alanine aminotrasferase (ALT) 77 U/L (normal 5-40), creatinine kinase (CK) 595 U/L (normal 40-150), lactate dehydrogenase (LDH) 556 U/L (normal 200-460), C-reactive protein (CRP) 560 nmol/L (normal <47.6); troponin I was negative. A repeat CT scan was performed which was demonstrated some alterations in the density of the mesenteric fat (misty mesentery) suggestive of pancreatic cancer progression. Soft tissue ultrasound examination of his left forearm flexors and right major pectoral muscle, showed findings consistent with myositis. Antineural antibodies were negative and sensory neuropathy was excluded by nerve conduction velocity testing. Electromyographic findings revealed a myopathic pattern with spontaneous activity. Biopsy of the right quadriceps muscle showed diffuse degenerative changes along with lymphocytic infiltrate and angular fibers. There was also thickening of the fibrous connective tissue septa and foci of fat infiltration; all the above findings were suggestive of myositis (Figure 1). Immunohistochemical stainining for CD8 expressing lymphocytes revealed sparse infiltrates of intramysial CD8-positive cells (brown diaminobenzidine staining). (Figure 2).

**Figure 3:**
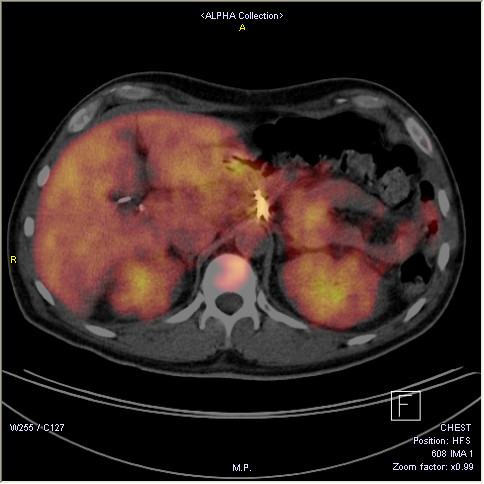
**Abdominal PET/CT**. Figure 2. PET/CT fusion image taken six months into treatment with erlotinib and corticosteroids showing postoperative findings only. There is no increased uptake of **^18^**F-FDG.

Intravenous methylprednisolone was initiated at a dose of 125 mg daily for two days and then was immediately switched to oral methylprednisolone 16 mg daily. A dramatic clinical response was observed shortly after treatment with glucocorticoids and the patient became fully ambulatory. Erlotinib 100 mg daily was started on standard gemcitabine based chemotherapy. Following his discharge, treatment with methylprednisolone 16 mg daily on tapering dose was continued for six months along with gemcitabine and erlotinib with complete remission of polymyositis. Re-evaluation by computed tomography (CT)-positron emission tomography-computed tomography (PET/CT) of the abdomen at that stage showed complete cancer remission (Figure 3). Chemotherapy was ceased along with corticosteroids and the patient remained on treatment with maintenance erlotinib remaining in good health and leading an active life. Unfortunately twelve months following his discharge a repeat CT scan showed pancreatic cancer relapse and prompted 2^nd ^line chemotherapy with gemcitabine. Interestingly, the progressive malignant disease was not followed by a myositis relapse.

## Discussion

Cancer of the pancreas is associated with several paraneoplastic syndromes. Polymyositis/dermatomyositisis is an inflammatory connective tissue disease predominantly involving skeletal muscle and is strongly associated with malignancy[5,6]. Hill *et al*[6] identified that 137 out of 914 cases of polymyositis had cancer, and reported that the standardized incidence ratio - SIR was 1.4 (95% CI 1.0-1.8) for men and 1.2 (0.9-1.6) for women. Polymyositis was associated with a raised risk of non-Hodgkin lymphoma, lung and bladder cancers, but not of pancreatic cancer. Sigurgeirsson *et al*[2] further sustained that inflammatory myopathy is strongly associated with malignancy and the malignant diseases most associated with inflammatory myositis were, in descending order: lung cancer, rectum and colon cancer, pancreatic cancer, kidney cancer, stomach cancer, breast cancer and Carpenter *et al*[3] reported poor prognosis when significant weakness is experienced at presentation.

Our patient was diagnosed with high grade, pancreatic-tail adenocarcinoma and six months following diagnosis and surgical resection, presented with proximal symmetrical muscle weakness, myalgias and generalised oedema while on treatment with gemcitabine. These signs were alarmingly suspicious of inflammatory myopathy and prompted further specific investigations. Eventually, diagnosis was confirmed with blood biochemistry, electromyography and muscle histology on the basis of a highly compatible history and physical findings.

According to Bohan and Peter's criteria[5], polymyositis is an inflammatory myopathy with no rash. It is defined by symmetric proximal muscle weakness, elevated serum muscle enzymes, myopathic changes on electromyography, characteristic muscle biopsy abnormalities and the absence of histopathologic signs of other myopathies. Muscle weakness is indeed the most common presenting feature of polymyositis. The onset is usually insidious and the distribution of weakness is typically symmetric and proximal. Myalgias occurs in less than 30% of the patiens[7].

Serum muscle enzyme levels are usually elevated in patients with polymyositis including CK, LDH, aldolase, AST and ALT which are routinely measured in the evaluation of myopathy. Although most patients with polymyositis have increased CK levels, reported series include patients with normal CK levels at presentation[8]. Specific autoantibodies such as those directed against cytoplasmic RNA synthetases, other cytoplasmic proteins, ribonucleoproteins, and certain nuclear antigens play important role in the assessment of patients with polymyositis given that they occur in approximately 30 percent of patients with polymyositis[9]. In addition to these, a novel autoantibody to a 155 kd protein (anti-p155) is commonly found in cancer associated dermatomyositis, but not in cancer associated polymyositis[10].

Even in clinical scenarios consistent with polymyositis, muscle biopsy is essential to establish the diagnosis. Typically, the cellular infiltrate is predominantly within the fascicle with inflammatory cells invading individual muscle fibers. Abnormal muscle fibers are scattered throughout the fascicle. Furthermore, there is evidence of cell-mediated immune mechanisms with presence of cytotoxic CD8+ T cells, which recognize antigens on the muscle fiber surface, and enhanced expression of major histocompatibility complex (MHC) antigens by the muscle fibers[11].

Although the scenario of gemcitabine induced myositis cannot be entirely excluded in our case, this appears to be highly unlikely; we found just one case report in the literature[12] implicating gemcitabine given along with docetaxel in inflammatory myopathy in a patient with lung adenocarcinoma and nevertheless as myositis in our patient heralded disease progression, it was fair to assume that it was disease rather than drug induced and thus necessitated standard immunosuppressive treatment along with cancer specific therapy. Subsequently, a dramatic clinical response was observed and the patient was ambulatory and fully recovered. Biochemical parameters normalised shortly after treatment with intravenous glucocorticoids and maintenance treatment with oral corticosteroids coupled with gemcitabine and erlotinib conferred complete response.

Glucocorticoids are indeed the cornerstone of initial therapy for polymyositis but in severely ill patients, azathioprine or methotrexate is preferable. The treatment of choice is prednisone in tapering doses for approximately one year, depending upon patient's response to therapy and achievement of disease control. Nevertheless, as many as 50% of patients with polymyositis do not respond to glucocorticoid therapy alone[2,13]. However in paraneoplastic polymyositis it is difficult to evaluate whether the clinical response of myositis is due to glucocorticoids *per se *or to the concurrent use of immunosuppressive chemotherapy or due to a synergistic effect among them.

Giving an insight in the pancreatic tumor cell, there are several immunogenic tumor antigens to elicit cellular as well as humoral immunity. In the Okada *et al *study[14], two DNA mismatch repair enzymes, Homo sapiens mutS homolog 2 (hMSH2) and Homo sapiens postmeiotic segregation increased 1 (hPMS1) were found to over-express in pancreatic ductal adenocarcinoma, and their antibodies were detected in sera from patients with pancreatic ductal adenocarcinoma, and in sera from patients with polymyositis, but not in sera from healthy individuals. Therefore, hMSH2 and hPMS1 could be immunogenic antigens in patients with pancreatic adenocarcinoma. Additionally Egberts *et al *showed that dexamethasone treatment had profound influence of pancreatic duct adenocarcinoma cells *in vitro *in terms of inhibition of invasiveness and activation of NFκB which was approved *in vivo *by reduced metastasing capability and reduced size of local tumour recurrence in an experimental model of pancreatic cancer[15]. These observations indicate that the link between malignancy and inflammatory myopathy relates possibly to the expression of common autoantigens between cancer tissue and muscle tissue in some patients with polymyositis. From a therapeutic point of view treatment with glucocorticoids should be justified in confirmed cases of polymyositis associated with pancreatic cancer along with cancer specific therapy. In addition our experience reinforces the basis for adjuvant immunotherapy as a potential additional therapeutic modality for all patients with pancreatic cancer, a notion which has been based mainly on experimental findings but appears to collect clinical substantiation and certainly merits further investigation.

## Conclusions

Polymyositis is a paraneoplastic syndrome which may be encountered in patients with pancreatic cancer causing significant morbidity. Clinicians should be aware of this syndrome as it may manifest at any stage during the physical history of pancreatic cancer. In this clinical setting a trial with corticosteroids along with cancer specific treatment may be of benefit.

## Competing interests

The authors declare that they have no competing interests.

## Authors' contributions

JS, GK and IDX were involved in the direct care of the patient, reviewed the literature and drafted the manuscript; MNG and AP are involved in the direct care of the patient, GA performed pathology, NT is involved in the direct care of the patient, coordinated the study and critically revised the manuscript. All authors read and approved the final manuscript.

## Consent

Written informed consent was obtained from the patient for publication of this case report and any accompanying images. A copy of the written consent is available for review by the Editor-in-Chief of this journal.

## Pre-publication history

The pre-publication history for this paper can be accessed here:

http://www.biomedcentral.com/1471-230X/11/33/prepub
